# *Clostridium botulinum* C3 Toxin for Selective Delivery of Cargo into Dendritic Cells and Macrophages

**DOI:** 10.3390/toxins14100711

**Published:** 2022-10-18

**Authors:** Maximilian Fellermann, Mia Stemmer, Reiner Noschka, Fanny Wondany, Stephan Fischer, Jens Michaelis, Steffen Stenger, Holger Barth

**Affiliations:** 1 Institute of Experimental and Clinical Pharmacology, Toxicology and Pharmacology of Natural Products, University of Ulm Medical Center, 89081 Ulm, Germany; 2 Institute for Medical Microbiology and Hygiene, University of Ulm Medical Center, 89081 Ulm, Germany; 3 Institute of Biophysics, Ulm University, 89081 Ulm, Germany

**Keywords:** clostridial C3 toxin, *Clostridium botulinum*, C3bot, C3bot_E174Q_, dendritic cells, macrophages, monocytes, stimulated emission depletion (STED), super-resolution microscopy

## Abstract

The protein toxin C3bot from *Clostridium botulinum* is a mono-ADP-ribosyltransferase that selectively intoxicates monocyte-derived cells such as macrophages, osteoclasts, and dendritic cells (DCs) by cytosolic modification of Rho-A, -B, and -C. Here, we investigated the application of C3bot as well as its non-toxic variant C3bot_E174Q_ as transporters for selective delivery of cargo molecules into macrophages and DCs. C3bot and C3bot_E174Q_ facilitated the uptake of eGFP into early endosomes of human-monocyte-derived macrophages, as revealed by stimulated emission depletion (STED) super-resolution microscopy. The fusion of the cargo model peptide eGFP neither affected the cell-type selectivity (enhanced uptake into human macrophages ex vivo compared to lymphocytes) nor the cytosolic release of C3bot. Moreover, by cell fractionation, we demonstrated that C3bot and C3bot_E174Q_ strongly enhanced the cytosolic release of functional eGFP. Subsequently, a modular system was created on the basis of C3bot_E174Q_ for covalent linkage of cargos via thiol–maleimide click chemistry. The functionality of this system was proven by loading small molecule fluorophores or an established reporter enzyme and investigating the cellular uptake and cytosolic release of cargo. Taken together, non-toxic C3bot_E174Q_ is a promising candidate for the cell-type-selective delivery of small molecules, peptides, and proteins into the cytosol of macrophages and DCs.

## 1. Introduction

C3bot is a protein toxin produced by *Clostridium botulinum* type C (*C. botulinum*) with a molecular weight of 23.5 kDa [1]. When C3bot reaches the cytosol of a target cell, it specifically mono-ADP-ribosylates Rho A, -B, and -C (in the following abbreviated as Rho) at position N41 [2,3,4]. For enzyme activity of C3bot, the amino acid E174 (without signal sequence) is essential, and the mutation E174Q leads to the enzymatically inactive and thus non-toxic variant C3bot_E174Q_ [5,6]. As the first C3 toxin identified, C3bot is the prototype of the C3-like ADP-ribosyltransferase family [7], comprising at least nine different members produced by different organisms (*C. botulinum*, *C. limosum*, *Staphylococcus aureus*, *Bacillus cereus*, and *Paenibacillus larvae*) [1,7,8,9,10,11,12,13,14,15]. In contrast to classical AB-type protein toxins, most bacterial C3 toxins (except for PlxA and C3larvin [9,16,17]) do not comprise a binding and translocation B-component, resulting in poor uptake into most cell types [2,18]. However, it was demonstrated that the clostridial C3 toxins are efficiently internalized into monocytes, macrophages, osteoclasts, and neurons [18,19,20,21,22]. Recently, human monocyte-derived dendritic cells (DCs) were also identified as specific target cells for the clostridial C3 toxins [23]. The C3 catalyzed Rho-modification in these cells results in impaired cell functions such as adhesion [24,25]; endo-, phago-, or exocytosis [2,26,27,28]; cell migration [20,21,29,30]; or differentiation/maturation [19] (for a review, see [2,31]). Notably, inactivation of Rho also results in a characteristic change in cell morphology as determined by formation of long protrusions [32,33]. Despite the effect of Rho-ADP-ribosylation is well examined, the exact cell entry mechanism of the C3 toxins remains widely unknown. Comparable to bacterial AB toxins, the clostridial C3 toxins are also internalized into early endosomes of macrophages and immature or mature DCs [18,23], but the exact mechanism of endosomal escape is not understood.

Since C3bot is by nature cell-type-selective for monocyte-derived cells, its non-toxic variant C3bot_E174Q_ represents a promising candidate as drug delivery tool. Cargo molecules can be attached to C3bot_E174Q_ and specifically delivered into monocytic cells via the C3bot uptake mechanism without harming the cells. This strategy has already been examined in first attempts by delivering reporter enzymes into the cytosol of macrophages [34,35,36]. Two of the tested enzymes were subunits of other bacterial toxins, i.e., DTA (21 kDa) from diphtheria toxin [35] or C2I (50 kDa) from the binary *C. botulinum* C2 toxin [36]. Moreover, RNase A (14 kDa) [34] was delivered into the cytosol of macrophages by C3bot_E174Q_. These cargo enzymes were attached via two strategies, i.e., generation of fusion proteins (C3bot_E174Q__C2I) [36] or by creating a modular system on the basis of the interaction of biotin (attached to cargo) and streptavidin (attached to C3bot_E174Q_) as proven for delivery of DTA and RNase A [34,35]. Despite this strategy seeming quite promising for delivering foreign proteins into macrophages, non-covalent attachment of cargo via the streptavidin–biotin system comes with some disadvantages. The preparation of the streptavidin-based transporters was contaminated by unspecific side-products [34], and streptavidin reduces the cell type-selectivity of the C3bot_E174Q_ transporters, potentially by influencing the interaction with cell surface proteins [34,35].

In the present study, we aimed to investigate the functions of C3bot and C3bot_E174Q_ as cellular delivery systems in more detail and to extend their applications. Therefore, a fusion protein based on C3bot and the green fluorescent protein (eGFP) was generated. This fusion protein was cell-type selectively internalized into human macrophages as compared with lymphocytes of the same donor ex vivo. Fusion to C3bot_E174Q_ strongly enhanced the internalization of the cargo model eGFP into early endosomes and cytosolic release of the cargo protein, as shown for macrophages and human DCs. Notably, fluorescence of the cytosolic eGFP was detected using a cell-fractionation assay verifying functionality of the released cargo. Moreover, on the basis of thiol–maleimide click chemistry, a novel modular system for fast, covalent, and specific attachment of cargo was created. Effective loading of the system with different cargo molecules was confirmed. It was demonstrated that the uptake of small molecules into human macrophages and DCs is strongly enhanced by their attachment to C3bot_E174Q_. Finally, the novel C3bot_E174Q_-based transport system enhanced the delivery of C2I into the cytosol of DCs and thereby proves the concept of the modular system with an established reporter enzyme. In conclusion, the C3bot_E174Q_ transport system can increase the cell membrane penetration of cargo molecules. Notably, C3bot is by nature cell type selective for monocyte-derived immune cells and is therefore the ideal delivery system for targeting macrophages and DCs.

## 2. Results

### 2.1. C3bot and C3bot_e174q_ Enhanced the Endosomal Uptake of eGFP

**Figure 1:**
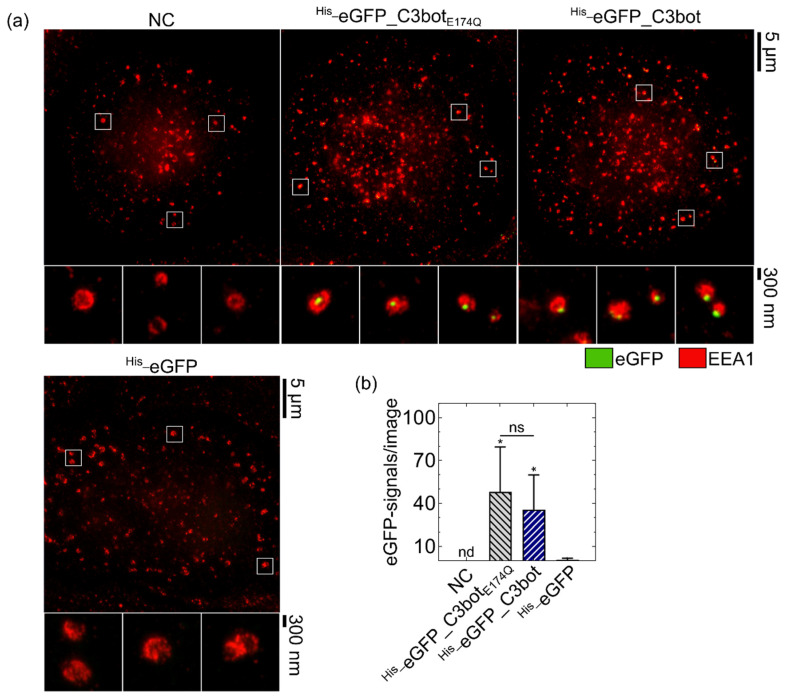
C3bot and C3bot_E174Q_ facilitated the endosomal uptake of eGFP into human monocyte-derived macrophages ex vivo. Cells were treated at 37 °C for 30 min with 250 nM ^His_^eGFP_C3bot, ^His_^eGFP_C3bot_E174Q_, or ^His_^eGFP, or were left untreated (NC). After staining of EEA1 and eGFP, STED super-resolution microscopy was performed. (**a**) Representative super-resolution images are depicted with magnification marked by white squares. Scale bars on the right correspond to 5 µm or 300 nm and hold for all images. (**b**) The experiment was repeated with macrophages derived from five individual and independent donors (n = 5 donors). The detected eGFP signals were quantified for each treatment, and the means are depicted with standard deviations (± SD). For NC, no eGFP signals were detectable, as marked by “nd”. Compared to the ^His_^eGFP samples, significance testing was performed using Student’s *t*-test (ns = not significant, * *p* < 0.05). By comparing the samples of ^His_^eGFP_C3bot_E174Q_ to ^His_^eGFP_C3bot, no significant differences were found, as indicated by “ns” and the line above the respective columns. The figure is modified from [37] under the authors’ rights.

To demonstrate that C3bot serves as a delivery tool, the uptake of the model cargo eGFP into its target cells was investigated. The peptide eGFP was genetically fused to C3bot or C3bot_E174Q_, and the uptake into human monocyte-derived macrophages analyzed by stimulated emission depletion (STED) super-resolution microscopy (Figure 1). Co-staining of the early endosomal antigen 1 (EEA1) was used as a marker for endosomes. The fusion proteins ^His_^eGFP_C3bot and ^His_^eGFP_C3bot_E174Q_ were both internalized into early endosomes, as indicated by surrounding of the eGFP-signals with EEA1 (Figure 1a).

These eGFP signals are condensed at specific locations within the endosomes either in the inner lumen or at the membrane of the vesicles (see magnifications). For ^His_^eGFP_C3bot_E174Q,_ 81.5 ± 15.9% and for ^His_^eGFP_C3bot 91.6 ± 4.5% of the detected eGFP signals were in close proximity (<250 nm) with EEA1 (see Table S2), indicating that most eGFP signals are associated with early endosomes. In contrast, ^His_^eGFP alone was not efficiently endocytosed by the macrophages, and only a neglectable amount of green fluorescence was detected, as shown in the quantification of the STED images (Figure 1b). All tested five blood monocyte donors showed an enhanced uptake of ^His_^eGFP_C3bot_E174Q_ and ^His_^eGFP_C3bot compared to ^His_^eGFP alone; however, the mean number of eGFP signals per image varies from donor to donor (see Table S1). These results thereby indicate that cellular uptake of eGFP is specific and significantly enhanced by coupling the cargo model to the transporter platform C3bot or C3bot_E174Q_.

### 2.2. ^His_^eGFP_C3bot_E174Q_ Was Selectively Internalized into Human Macrophages Ex Vivo

**Figure 2:**
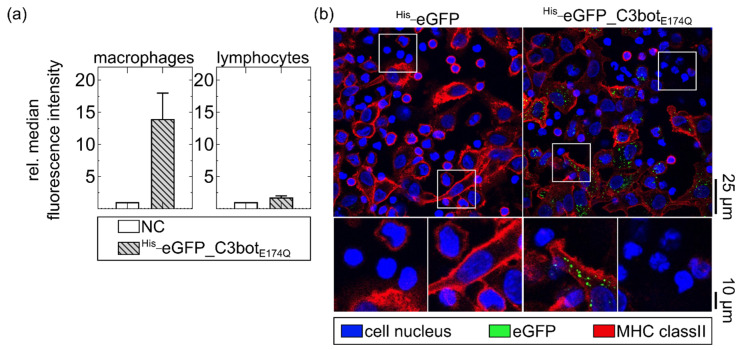
Cell-type-selective uptake of ^His_^eGFP_C3bot_E174Q_ into primary human macrophages compared to lymphocytes ex vivo. (**a**) Human-monocyte-derived macrophages and lymphocytes of the same donor were separately treated with 800 nM ^His_^eGFP_C3bot_E174Q_ for 20 min at 37 °C. The cells were washed with PBS, and extracellular or membrane-bound eGFP signals were quenched with trypan blue directly before flow cytometry measurement. The averaged relative median fluorescence intensity (normalized to untreated cells (NC)) is depicted in the column diagrams (mean ± SD, n = 5). (**b**) Monocyte-derived macrophages and lymphocytes of the same human blood donor were co-cultured (1:5 ratio) and treated with 400 nM ^His_^eGFP_C3bot_E174Q_ or ^His_^eGFP at 37 °C for 1 h. The cells were fixed, and the cell nuclei (blue) were stained with DAPI. MHC class II (red) was stained as marker for macrophages. All in confocal microscopy detected fluorescence signals (eGFP, DAPI, and MHC class II) were merged, and representative images are depicted with magnification as indicated by the white squares. Scale bars on the right apply to the complete row of images (25 µm for full-size images, and 10 µm for magnifications). The figure is modified from [37] under the authors’ rights.

After confirming that transport of cargo into macrophages can be enhanced by C3bot or C3bot_E174Q_, we investigated whether ^His_^eGFP_C3bot_E174Q_ still possessed the cell-type selectivity of wild-type C3bot. Hence, cellular uptake into human-monocyte-derived macrophages was compared with uptake into lymphocytes of the same blood donor. Initially, the different cells were treated separately with ^His_^eGFP_C3bot_E174Q_, and internalization of eGFP was quantified in flow cytometry (Figure 2a). Since only internalized cargo should be analyzed, cell-surface-bound eGFP signals were quenched by using an established trypan-blue-based assay [38,39,40]. Uptake of ^His_^eGFP_C3bot_E174Q_ into macrophages was strongly enhanced in comparison with lymphocytes, which internalized only a minor portion of the fusion protein (Figure 2a). Notably, even with 800 nM of ^His_^eGFP_C3bot_E174Q_, only a minor portion was internalized by the lymphocytes (Figure 2a; for the experiment with a lower concentration, see Figure S1).

On the basis of these findings, a co-culture of macrophages and lymphocytes of the same blood donor was treated with ^His_^eGFP_C3bot_E174Q_. For the control, the co-culture was treated with ^His^-eGFP alone. The macrophage population was marked by staining of the major histocompatibility complex (MHC) class II on the cell surface (Figure 2b). In this co-culture, ^His_^eGFP_C3bot_E174Q_ was cell-type selectively internalized into human macrophages, as indicated by the green signals surrounded by red MHC class II staining. Moreover, about 70–90% of the total macrophage population internalized ^His_^eGFP_C3bot_E174Q_ (see Figure S2). Hence, direct coupling of cargo molecules to the transporter system did not affect the cell-type selectivity, and C3bot_E174Q_ can be used to enhance the uptake of cargo molecules.

### 2.3. Functional eGFP Was Delivered into the Cytosol of DCs and Macrophages via C3bot and C3bot_E174Q_

**Figure 3:**
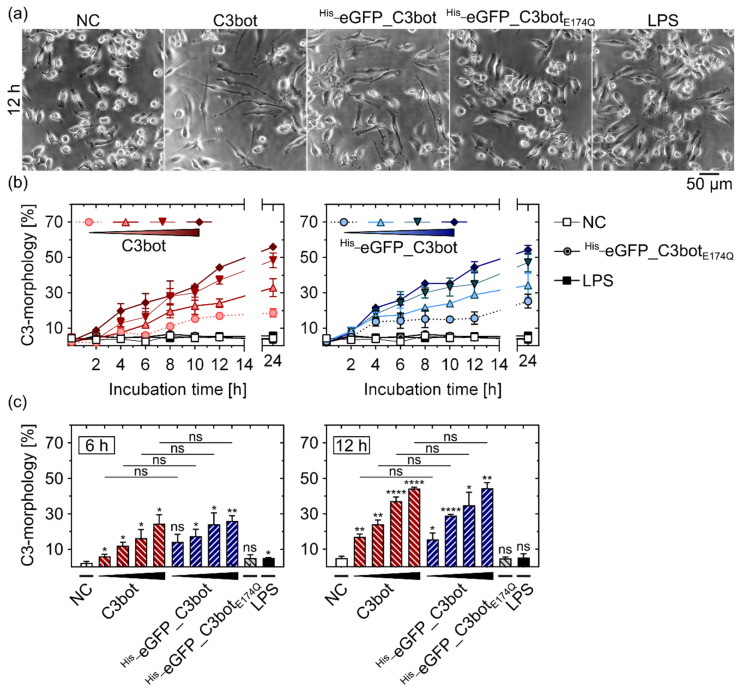
Fusion of eGFP to C3bot did not affect the cytosolic release of C3bot in J774A.1 macrophages. (**a**) J774A.1 cells were treated with C3bot, ^His_^eGFP_C3bot, ^His_^eGFP_C3bot_E174Q_ (200 nM each), or 1 µg/mL LPS, or were left untreated (NC). The images show representative phase contrast images after an incubation time of 12 h with 50 µm scale bar. (**b**,**c**) J774A.1 macrophages were treated with increasing concentrations (25, 50, 100, 200 nM) of C3bot or ^His_^eGFP_C3bot, with 200 nM ^His_^eGFP_C3bot_E174Q_, with 1 µg/mL LPS, or were left untreated (NC). Phase contrast images were taken after incubation times of 2, 4, 6, 8, 10, 12, and 24 h at 37 °C. The cell portion with characteristic stellate C3 morphology was quantified and divided by the total amount of cells per image. The ratios (mean ± SD, n = 3) are depicted in time course diagrams (**b**) and column diagrams after 6 or 12 h (**c**). (**c**) Compared to the NC, statistical significance was tested via Student’s *t*-test, and columns are labeled according to following significance levels: ns *p* > 0.05, * *p* < 0.05, ** *p* < 0.01, **** *p* < 0.0001. Additionally, the same concentrations of C3bot and ^His_^eGFP_C3bot were compared via Student’s *t*-test, as indicated by the lines and significance levels above the representative columns. The figure is modified from [37] under the authors’ rights.

Next, we investigated whether the cargo model eGFP was released into the cytosol of target cells. Since eGFP is hardly detectable in the cytosol, even with state-of-the-art fluorescence microscopic techniques, at first, an indirect approach based on the cytotoxic effect of C3bot was used. We compared the morphological changes induced by C3bot with those induced by cargo-labeled ^His_^eGFP_C3bot to evaluate whether coupling of cargo interfered with the uptake process of the toxin. Therefore, cells of a mouse macrophage cell line (J774A.1) were treated with increasing concentrations of C3bot or ^His_^eGFP_C3bot (Figure 3). Treatment with ^His_^eGFP_C3bot induced characteristic changes in cell macrophage morphology, i.e., formation of long cell protrusions, comparable to wild-type C3bot (Figure 3a). In contrast, ^His_^eGFP_C3bot_E174Q_ did not induce such characteristic changes in cell morphology, confirming that enzymatic activity of C3bot is required for this effect. Since the fusion proteins were produced in *Escherichia coli* (*E. coli*), the effect of lipopolysaccharides (LPS) was also tested. LPS endotoxins are common impurities in recombinant protein expression in Gram-negative bacteria that can activate immune cells, e.g., macrophages [41,42]. Despite treatment with LPS slightly influencing J774A.1 cell morphology and more intracellular inclusions being visible compared to the negative control (NC), the clear and striking formation of protrusions cannot be explained by a potential LPS contamination (Figure 3a). Hence, the results indicate that active C3bot is required for induction of the morphological change and, most importantly, eGFP labeling did not affect this effect. This becomes even more obvious by quantifying the cells with C3 morphology and comparing different concentrations in a time course (Figure 3b) or at defined time points (Figure 3c). Importantly, no significant differences were detected by comparing same concentrations of wild-type C3bot with ^His_^eGFP_C3bot.

**Figure 4:**
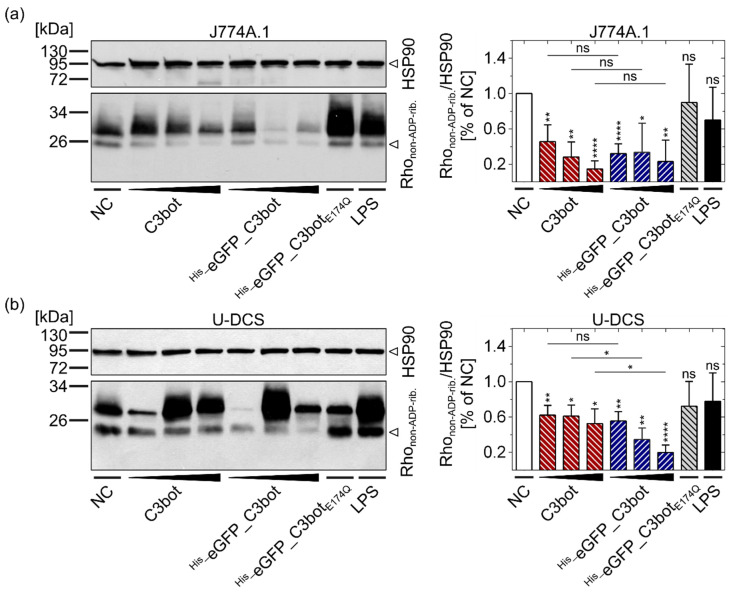
Fusion of eGFP to C3bot did not affect the cytosolic ADP-ribosylation of Rho in J774A.1 macrophages or U-DCS cells. J774A.1 cells (**a**) and U-DCS cells (**b**) were treated with increasing concentrations (50, 100, 200 nM) of C3bot or ^His_^eGFP_C3bot, respectively, with 200 nM ^His_^eGFP_C3bot_E174Q_, with 1 µg/mL LPS, or they were left untreated (NC). After 7 h of incubation at 37 °C, the cells were washed and lysed, and then a sequential ADP-ribosylation assay was performed for detection of non-ADP-ribosylated Rho in intact cells (lower band in left lower panels). The relative integrated density values for Rho_non-ADP-rib._ were normalized to the HSP90 loading control (left upper panels) and depicted in a column diagram (mean ± SD, right panels). The detected bands above 26 kDa (upper band in left lower panels) were probably unspecific binding sites of the used streptavidin–peroxidase conjugate, which did not correlate with any treatment conditions. For J774A.1 macrophages in (**a**), five replicates (n = 5), and for U-DCS cells in (**b**), four replicates (n = 4) were averaged. Notably, weak signals in the sequential ADP-ribosylation assay indicated strong toxin activity in intact cells. Compared to the NC, statistical significance was tested via Student’s *t*-test, and columns are labeled according to following significance levels: ns *p* > 0.05, * *p* < 0.05, ** *p* < 0.01, **** *p* < 0.0001. Additionally, the same concentrations of C3bot and ^His_^eGFP_C3bot were compared via Student’s *t*-test, as indicated by the lines and significance levels above the representative columns. The figure is modified from [37] under the authors’ rights.

These results were furthermore confirmed by analyzing the Rho ADP-ribosylation status inside the cells. A sequential Rho ADP-ribosylation assay was used for detection of non-ADP-ribosylated Rho in intact cells (Rho_non-ADP-rib._) (Figure 4). Notably, in this assay, weak signals indicate strong ADP-ribosylation of Rho by the toxin inside the target cells. Equal protein loading was controlled by detection of heat shock protein 90 (HSP90). The detected Rho_non-ADP-rib._ decreased, i.e., Rho-ADP-ribosylation in the intact cells increased with the concentration of C3bot or ^His_^eGFP_C3bot. In accordance with the morphological assays, eGFP labeling inhibited neither the Rho ADP-ribosyltransferase activity of C3bot in J774A.1 macrophages (Figure 4a) nor the activity in human DCs derived from a sarcoma cell line (U-DCS cells, see Figure 4b). Taken together, the results indicate that cytosolic release of C3bot into target cells (macrophages and DCs) is not inhibited by fusion with the cargo model eGFP.

Since this approach is quite indirect for the detection of cytosolic cargo release, alongside the fact that intracellular degradation of the fusion constructs cannot be excluded, we directly analyzed the presence of cytosolic eGFP with an established digitonin-based cell fractionation [34,36]. After treatment with the eGFP-labeled proteins (^His_^eGFP, ^His_^eGFP_C3bot, and ^His_^eGFP_C3bot_E174Q_), the cells were separated into cytosolic and membrane fractions.

**Figure 5:**
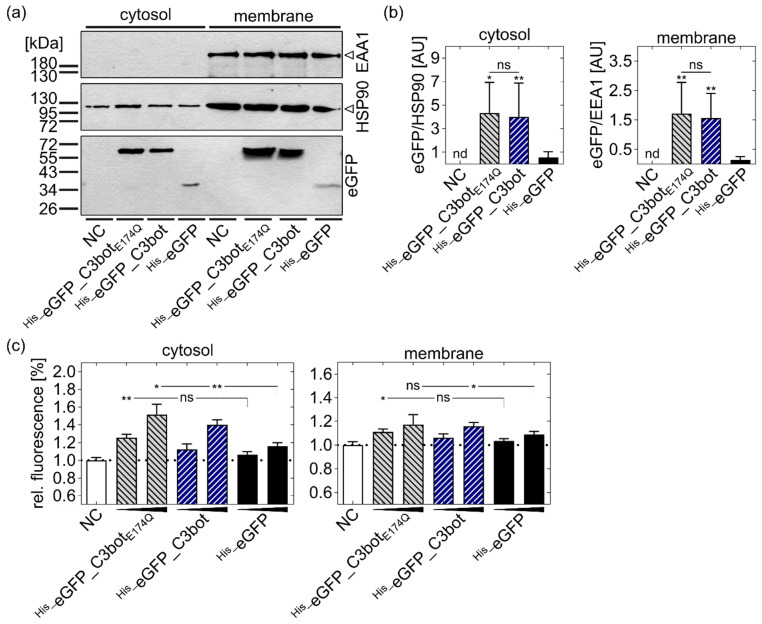
C3bot and C3bot_E174Q_ increased the cytosolic release of the functional cargo model eGFP. (**a**,**b**) U-DCS cells were treated with ^His_^eGFP_C3bot, ^His_^eGFP_C3bot_E174Q_, or ^His_^eGFP (250 nM each), or were left untreated (NC) for 1 h at 37 °C. Subsequently, the cytosol was separated from the membrane fraction (containing endosomes) in a digitonin-based assay. (**a**) In a Western blot, the separation was confirmed by detection of EEA1 as a maker for endosomes that should not be present in the cytosolic fraction. HSP90 was detected as a cytosolic marker, present in both fractions, since the cytosol did not completely leave the cells (see Section 5.14). The presence of eGFP in the cytosol or membrane fraction was analyzed by using a primary anti-GFP antibody. (**b**) The Western blots were quantified, and the normalized integrated density values were plotted in a column diagram as mean ± SD (n = 7). For the cytosolic fraction, eGFP was normalized to the detected HSP90 signals, and for the membrane fraction, it was normalized to the EEA1 signals. The individual values for each repetition are available in the Supplementary Materials (Table S3 for the cytosolic fraction and Table S4 for the membrane fraction). (**c**) U-DCS cells were treated with ^His_^eGFP_C3bot, ^His_^eGFP_C3bot_E174Q_, or ^His_^eGFP (250 and 500 nM each), or left untreated (NC) for 5 h at 37 °C. Digitonin-based cell fractionation was performed as described above and controlled by Western blotting (data not shown). The functionality of eGFP in each fraction (cytosol in the left panel and membrane in the right panel) was analyzed by fluorescence detection at 488 nm and an emission wavelength of 510 nm in a microplate reader. (**b**,**c**) Compared to the respective ^His_^eGFP treatment, statistical significance was tested via Student’s *t*-test, and the following significance levels were defined: ns *p* > 0.05, * *p* < 0.05, ** *p* < 0.01. (**b**) Additionally, the same concentrations of C3bot and ^His_^eGFP_C3bot were compared via Student’s *t*-test, as indicated by the lines and significance levels above the representative columns.

For isolation of the cytosol, digitonin was used to form small pores into the cell membrane that are smaller than endosomes or other organelles. Hence, only the cytosolic proteins were able to leave the cells. Successful separation of the cytosolic fraction from the membrane fraction (containing endosomes) was confirmed by Western blot detection of EEA1, which was only present in the membrane fractions (Figure 5a). As a loading control for cytosolic proteins, HSP90 was detected.

Already after 1 h of incubation with the constructs, signals for eGFP were detected in both fractions, indicating cytosolic release of eGFP (Figure 5a). For ^His_^eGFP_C3bot and ^His_^eGFP_C3bot_E174Q_, the eGFP signals were detected at a height of about 55 kDa, corresponding to the size of full-length fusion proteins (calculated at 52 kDa). For ^His_^eGFP, the signals were detected at about 34 kDa, which also fitted into the calculated molecular weight of about 28.4 kDa. These results indicate that eGFP-labeled proteins were not degraded during cellular uptake. Due to the nature of this assay, it is not possible to compare the protein amounts in one fraction with that in the other fraction, but rather the signals within one fraction should be compared. In both fractions, the detected eGFP signals were much stronger for the fusion constructs ^His_^eGFP_C3bot and ^His_^eGFP_C3bot_E174Q_ compared to ^His_^eGFP alone (Figure 5a), indicating that uptake into intracellular vesicles (membrane fraction) as well as cytosolic release of the cargo model eGFP were significantly enhanced by coupling to C3bot or C3bot_E174Q_ (for Western blot quantification and statistical analysis, see Figure 5b).

For assay control, it was confirmed that the fusion proteins ^His_^eGFP_C3bot and ^His_^eGFP_C3bot_E174Q_ were equally detected compared to ^His_^eGFP by the GFP antibody (see Figure S3b,c). In this additional Western blot (Figure S3b,c), the detected signals for ^His_^eGFP_C3bot were slightly weaker compared to ^His_^eGFP and ^His_^eGFP_C3bot_E174Q_; however, this minor difference was neglected since it did not object to the final conclusion in the digitonin-based cell fractionation assay.

After confirming that cargo delivery can be enhanced by coupling to C3bot and C3bot_E174Q_, we investigated whether the cytosolic eGFP was still functional. Therefore, fluorescence of the cytosolic and membrane fraction was analyzed with a microplate reader after treatment of cells with ^His_^eGFP, ^His_^eGFP_C3bot, or ^His_^eGFP_C3bot_E174Q_ and digitonin-based separation. Fluorescence signals thereby increased in a concentration-dependent manner, and cytosolic fluorescence was significantly enhanced by coupling eGFP to the C3bot transporters (Figure 5c, left panel). Taken together, the results indicated that the endosomal uptake and cytosolic release of the eGFP cargo model into macrophages and DCs can be strongly enhanced by using C3bot or C3bot_E174Q_ as delivery tools and that the cargo remains functional inside these target cells.

### 2.4. Characterization of a Modular System for Fast Attachment of Cargo to C3bot_E174Q_

After proofing the concept of using C3bot_E174Q_ as a shuttle system for the cargo model eGFP, the next step was to generate a modular delivery system for fast attachment of various cargos, i.e., small molecules and proteins. Since the earlier published streptavidin-biotin system comes with the cost of reduced cell type selectivity [34,35], direct coupling of C3bot_E174Q_ with cargo via thiol–maleimide click chemistry was investigated to possibly overcome this problem. By nature, C3bot_E174Q_ does not contain any thiol group (no cysteine), and therefore this reaction group can simply be inserted side specifically by a point mutation. A single cysteine amino acid was inserted at the N-terminal end of C3bot_E174Q_ (A1C), and this mutant will be further referred to as ^Cys^-C3bot_E174Q_. After protein purification, the thiol group can be loaded with different maleimide-labelled cargo molecules (see the cartoon in Figure S4a and example cargo in Figure S4b) by incubation for 2 h on ice. Successful protein loading was indicated by a shift to higher molecular weight, as observed by SDS-PAGE. As proof of concept, we loaded different maleimide-labeled cargos, i.e., a small molecule fluorophore (maleimide_Dylight 488 (mDL488), 0.8 kDa), larger fluorescein-isothiocyanate-labeled polyethylene glycol (mPEG_FITC, 5 kDa), or the reporter enzyme C2I (mC2I, 50 kDa). For all cargo molecules, a shift in molecular weight was detected as expected for successful coupling (Figure S4c).

**Figure 6:**
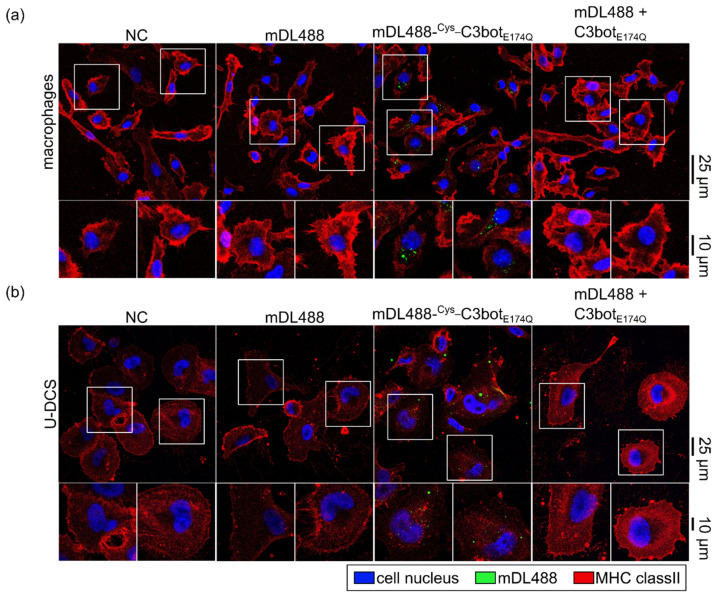
The small-molecule fluorophore was delivered into macrophages and DCs via ^Cys_^C3bot_E174Q_. Human-monocyte-derived macrophages (**a**) and U-DCS cells (**b**) were treated with free mDL488, mDL488-^Cys_^C3bot_E174Q_, or uncoupled mDL488 together with C3bot_E174Q_ (250 nM each), or were left untreated (NC) for 30 min at 37 °C. The cells were fixed, and cell nuclei (DAPI) and MHC class II were stained as markers for cell shape and size. Confocal microscopy was used to detect fluorescence signals, and the signals for mDL488 (green), DAPI (blue), and MHC class II (red) were merged. Representative images are depicted with magnification as indicated by the white squares. Scale bars on the right are applied for the complete row of images (25 µm for full-size images, and 10 µm for magnifications).

After confirming the cargo coupling to the ^Cys_^C3bot_E174Q_-system, successful cellular delivery was tested in different assays. First, the uptake of the small molecule fluorophore mDL488 into macrophages (Figure 6a) and DCs (Figure 6b) was investigated. Internalization of mDL488 was only facilitated when the cargo was directly coupled to ^Cys_^C3bot_E174Q_. In contrast, neither for the treatment with cargo alone (mDL488) nor in combination with free uncoupled C3bot_E174Q_ green internalization signals were detected. These results indicate that the generated modular system enhanced cellular uptake directly and not indirectly by simply enhancing endocytosis. Comparable results were obtained for the lager mPEG_FITC cargo model, which was also more efficiently internalized into target cells after coupling to ^Cys_^C3bot_E174Q_ (Figure S5).

**Figure 7:**
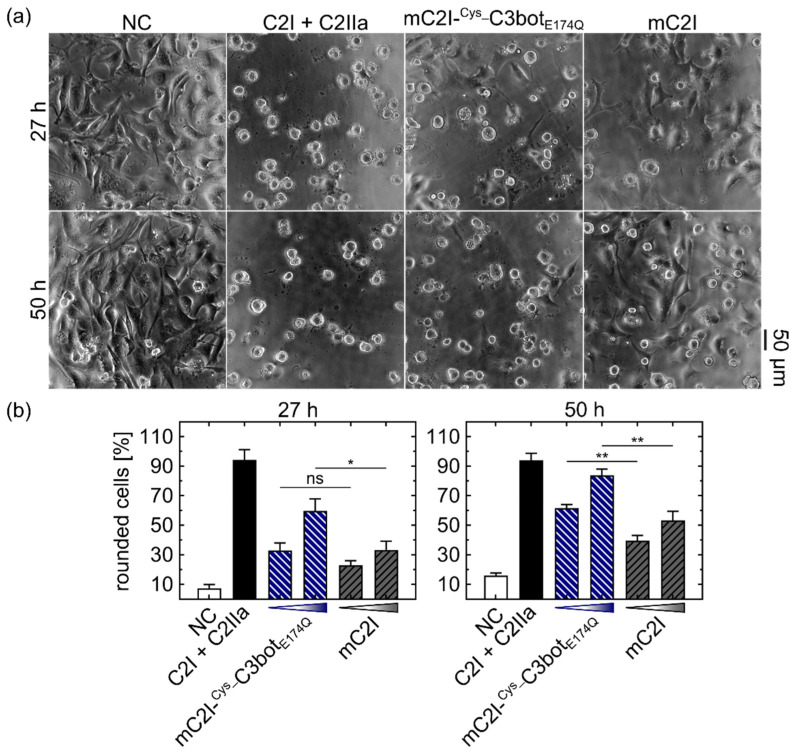
^Cys_^C3bot_E174Q_ enhanced the cytosolic release of the reporter enzyme C2I. (**a**) U-DCS cells were treated with 200 nM mC2I-^Cys_^C3bot_E174Q_, 880 nM mC2I, or a combination of 1 nM C2I with 1.66 nM C2IIa, or were left untreated (NC). Phase contrast images were taken after 27 and 50 h of incubation, and representative micrographs are shown with a 50 μm scale bar. (**b**) U-DCS cells were treated with 50 or 200 nM mC2I-^Cys_^C3bot_E174Q_, 220 or 880 nM mC2I, or a combination of 1 nM C2I with 1.66 nM C2IIa, or were left untreated (NC). Phase contrast images were taken after 27 and 50 h of incubation. The number of rounded cells was counted and divided by the total cell number, and the resulting ratios are depicted in column diagrams as mean ± SD (n = 3) for the indicated time points. The values for mC2I-^Cys_^C3bot_E174Q_ were compared with the corresponding mC2I concentrations, and statistical significance was tested via Student’s *t*-test (indicated with a line above columns) according to the following significance levels: ns *p* > 0.05, * *p* < 0.05, ** *p* < 0.01. The figure is modified from [37] under the authors’ rights.

Finally, we investigated whether the cytosolic release of cargo was also enhanced by the modular thiol–maleimide system. Therefore, the established reporter enzyme C2I was labeled with maleimide (mC2I) via the bifunctional linker m-maleimidobenzoyl-N-hydroxysuccinimide ester (MBS) and coupled to ^Cys_^C3bot_E174Q_. When C2I reached the cytosol, it ADP-ribosylated G-actin, leading to cell rounding without affecting the cell viability within 2 days of incubation [43,44]. Cell rounding also occurred when U-DCS cells were treated with C2I in combination with its activated B-component C2IIa, providing a robust and established control for successful cytosolic release of cargo (Figure 7a). In accordance with the literature [44,45], only minimal effects on cell viability/proliferation were detected for the delivered C2I (Figure S6).

Although mC2I alone applied in high concentrations (220 or 880 nM) for 27 or 50 h also induced some cell rounding of the dendritic sarcoma cells, this effect was significantly enhanced by coupling mC2I to ^Cys_^C3bot_E174Q_ (mC2I-^Cys_^C3bot_E174Q_), as shown in the quantification (Figure 7b). For proper control, 4.4-fold higher concentrations of mC2I were used in comparison to mC2I-^Cys_^C3bot_E174Q_ in order to compensate for free mC2I present in the coupling product (Figure S4b). Nevertheless, the ^Cys_^C3bot_E174Q_ transporter significantly increased the cytosolic release of mC2I, proving that the modular C3bot system can serve as selective and specific tool for delivery of cargo molecules into the cytosol of human monocytic cells.

## 3. Discussion

The protein toxin C3bot selectively enters the cytosol of monocytic cells, including macrophages and DCs, by endocytosis, and inhibits Rho-dependent processes in such cells. Thereby, the toxin down-modulates essential functions of these important immune cells, which should be detrimental in the context of an infection with C3-toxin-producing bacteria. On the other hand, the cell-type selectivity of the C3 toxin can be exploited for the targeted pharmacological down-modulation of excessive pro-inflammatory activity of macrophages in the context of traumatic diseases [21]. Since hyperactive macrophages and DCs play crucial roles in several inflammatory diseases, they are important drug targets, and the C3 toxin should be a promising compound to selectively and specifically suppress excessive reactions of these innate immune cells. However, the cell-type selectivity of C3bot towards monocytic cells can be exploited for pharmacological purposes in a second way, i.e., for targeted delivery of therapeutic (macro-) molecules into these target cells and their controlled release into the cytosol. Various bacterial AB-type protein toxins have been used as drug delivery systems because of their unique mode of action, i.e., endocytic uptake and endosomal release of the therapeutic cargo molecules to reach cytosolic drug targets [46,47,48]. Since C3bot by nature enters monocyte-derived cells, a non-toxic variant of C3bot should represent an ideal molecule for targeted drug delivery into macrophages and DCs. Potentially, the cell penetration of novel therapeutics, e.g., peptides, proteins, nucleic acids, or cell-membrane-impermeable small molecules, can be facilitated, and/or selective targeting of macrophages and DCs as important immune cells can be improved by using C3bot_E174Q_ as a drug delivery tool. The basic concepts for such a transport system were investigated in this study.

As proof of concept, the cargo model eGFP was genetically fused to both C3bot and C3bot_E174Q_, and cellular uptake of the resulting fusion proteins into target cells was investigated in comparison to non-target human blood cells. The fusion protein ^His_^eGFP_C3bot_E174Q_ was selectively internalized into the human-monocyte-derived macrophages much stronger compared to lymphocytes of the same blood donor. Hence, the cell-type selectivity of the wild-type C3bot was maintained, despite the attachment of eGFP. The fluorescent protein eGFP is commonly used to follow uptake processes or to localize intracellular proteins. The generated fusion proteins (^His_^eGFP_C3bot and ^His_^eGFP_C3bot_E174Q_) were specifically internalized into early endosomes of human-monocyte-derived macrophages, as unveiled by STED super-resolution microscopy. These results confirm earlier results obtained for the J774A.1 cell line [18] and provide more details about the localization within early endosomes. The eGFP signals are not evenly distributed over the complete vesicles, but rather are condensed at specific sites where potential translocation into the cytosol could take place. Earlier, our group observed a similar C3bot condensation in endosomes for human-monocyte-derived mature or immature DCs [23]. Only neglectable amounts of ^His_^eGFP alone were internalized into human-monocyte-derived cells, indicating that the C3bot transporters strongly enhance the cellular uptake of this cargo model. After endocytosis, wild-type C3bot is released into the cytosol of macrophages or DCs [18,23]. However, due to the stable β-barrel structure, eGFP can potentially hinder the translocation process, resulting in lower toxin activity, as shown for the diphtheria toxin [49] or for the anthrax pore [50]. In the present study, we investigated whether this was also the case for C3bot translocation by comparing wild-type C3bot with ^His_^eGFP_C3bot in intoxication assays. In contrast to the diphtheria toxin or the anthrax pore, not even the slightest reduction in toxin activity was detected for the fusion construct ^His_^eGFP_C3bot. Hence, this could be the result of (i) a very effective delivery of the cargo together with C3bot into the cytosol, or (ii) very effective cleavage of the fusion construct in the early endosomes to only release C3bot into the cytosol. This second possibility, however, was ruled out by detection of cytosolic full-length ^His_^eGFP_C3bot and ^His_^eGFP_C3bot_E174Q_ in a digitonin-based cell fractionation assay. Notably, no degradation products were detected in either the cytosol or the membrane fraction, excluding endosomal degradation. Importantly, cytosolic eGFP delivered by C3bot or C3bot_E174_ was also proven to be functional by fluorescence detection. Since hypothesis (ii) could be rejected, the possibility of (i) being correct seems plausible, indicating that even relatively stable proteins such as eGFP are efficiently delivered into the cytosol of target cells via the toxins’ uptake mechanism. It is possible that C3bot uses another translocation mechanism compared to the diphtheria or anthrax toxin. This could explain the preserved toxin activity of the eGFP fusion construct (^His_^eGFP_C3bot). This seems reasonable since C3bot has no B-component that remains inside the endosomes [2,18], while this is the case for the classical AB-type protein toxins. Moreover, it was shown earlier that C3bot does not form pores but rather induces membrane flickering upon acidification [18]. By using C3bot or C3bot_E174Q_ as a drug delivery tool, this special mechanism could potentially be utilized to deliver stable cargo molecules into the cytosol of C3bot-target cells. In the digitonin-based cell-fractionation assay, low amounts of cytosolic ^His_^eGFP were detected when the cells were treated with cargo alone. This minor portion can be explained by unspecific uptake and release. Alternatively, the His-tag could potentially function as a very inefficient cell-penetrating peptide, as shown for cationic elastin-like polypeptide tags that enhance the cellular uptake of GFP in dependency on the number of positive charges [51]. However, in contrast to cells treated with ^His_^eGFP_C3bot_E174Q_ or ^His_^eGFP_C3bot, this portion is neglectable, indicating that the C3bot-based transporters significantly enhanced the cytosolic release of cargo.

To evaluate drug delivery via C3bot in more detail, a modular system for fast attachment of cargo was generated on the basis of thiol–maleimide click chemistry. Three different cargo molecules were efficiently loaded onto the ^Cys_^C3bot_E174Q_-transporter, and uptake into DCs and macrophages was evaluated. Labeling with mDL488 was used to simulate small molecule cargo (0.8 kDa). A polyethylene glycol cargo model (mPEG_FITC) was used to simulate more bulky cargo molecules (5 kDa), and the reporter enzyme C2I was used as a model for larger protein cargo (50 kDa). For mDL488 and mPEG_FITC, uptake into macrophages and DCs was strongly enhanced by coupling to ^Cys_^C3bot_E174Q_. Increased cytosolic release into DCs was detected for mC2I. Thereby, the modular system was proven to be functional and can be used to deliver a wide range of different drugs into these target cells.

Besides the action on macrophages and DCs, earlier publications have shown that C3bot can independently of its enzymatic activity promote the outgrowth of neuronal cells after spinal cord injury [22]. Coupling C3bot_E174Q_ with novel drugs could potentially enhance this effect. Moreover, C3bot_E174Q_ could potentially also be used as a drug delivery tool for targeting neuronal cells upon local application. The thiol–maleimide system could serve as a platform for fast attachment of different drug candidates to C3bot_E174Q_ and thereby accelerate such a drug development process.

By comparing this thiol–maleimide system with the earlier published biotin–streptavidin coupling system [19,34] for coupling of cargo molecules to C3bot_E174Q_, this novel system provides some benefits but also comes with some costs. A disadvantage of the thiol–maleimide system is that the cargo molecules themselves should not contain thiol groups to ensure a specific and easy coupling process. Otherwise, maleimide-labeled cargo molecules could form multimers. This could be prevented by using protection groups, but this complicates the coupling process. In contrast, the main advantages of the thiol–maleimide system over the biotin–streptavidin coupling system are that streptavidin-induced multimerization can be excluded and that streptavidin cannot influence the natural cell-type selectivity of C3bot, which was reduced/lost, as shown in earlier publications for the biotin–streptavidin coupling system [34,35]. Internalization of the novel system only depends on ^Cys_^C3bot_E174Q_ and the attached cargo molecule.

Overall, the fully modular thiol–maleimide system will widely extend the applications of C3bot_E174Q_ as a cell-type-selective drug delivery system for monocyte-derived cells, as well as allowing for fast and easy attachment of various therapeutic cargo molecules including macromolecules such as enzymes, peptides, or nucleic acids.

## 4. Conclusions

Taken together, we demonstrated that C3bot and its non-toxic variant C3bot_E174Q_ can be used to deliver different cargo molecules into the cytosol of human macrophages and DCs. Direct fusion of eGFP as a cargo model to C3bot_E174Q_ neither influenced the cell-type selectivity as evaluated for human blood cells, nor interfered with cytosolic release of the C3 toxin. C3bot and C3bot_E174Q_ can therefore serve in the delivery of relatively stable functional proteins into the cytosol of their target cells. Additionally, thiol–maleimide technology was used for rapid and easy attachment of various maleimide-labeled cargo molecules to ^Cys_^C3bot_E174Q_ in order to generate a fully modular transport system for the targeted delivery and cytosolic release of molecules into macrophages and DCs.

## 5. Materials and Methods

### 5.1. Cell Culture

All cells were cultured at 37 °C at constant saturated humidity and 5% CO_2_ up to passage number 25. For U-DCS cells, a medium mix of IMDM (Lonza, Basel, Switzerland) and RPMI 1640 (Gibco-Life Technologies, Carlsbad, CA, USA) in 4:1 ratio was used, and it was supplemented with 10% fetal calf serum (Gibco-Life Technologies, Carlsbad, CA, USA), 0.1% insulin–transferrin–sodium selenite supplement (Roche Diagnostics, Basel, Switzerland), 1% L-glutamine (Thermo Fisher Scientific, Waltham, MA, USA), and 100 U/mL (1%) penicillin–streptomycin (Gibco-Life Technologies, Carlsbad, CA, USA). Subconfluent U-DCS cells were passaged after trypsin detachment (Roche Diagnostics, Basel, SUI) every 2 to 3 days and split into ratios of 1:2 or 1:3, respectively. J774A.1 macrophages were cultivated in DMEM with 10% FCS, 1 nM sodium pyruvate, 1% penicillin–streptomycin, and 1% non-essential amino acids (all Gibco-Life Technologies, Carlsbad, CA, USA). Subconfluent J774A.1 cells were passaged after mechanical detachment with a cell scraper (Sarstedt, Nümbrecht, Germany) every 2 to 3 days and split into ratios of 1:4 or 1:10, respectively. During passaging, cells were seeded in microtiter plates and used for the experiments on the following or next but one day, respectively.

### 5.2. Differentiation of Human Monocytes into Macrophages and Co-Cultivation with Lymphocytes

Density gradient centrifugation (Ficoll-Paque™ Plus; GE Healthcare, Chicago, IL, USA) was used to isolate human peripheral blood mononuclear cells (PBMCs) from buffy coat preparations of anonymous healthy donors (Institute of Transfusion Medicine, Ulm University). PBMCs were allowed to adhere in a cell culture flask for 90 min in AIM V cell culture medium (Gibco-Life Technologies, Carlsbad, CA, USA). Non-adherent autologous cells (lymphocytes) were transferred to a fresh flask and kept in AIM V until their use in the incubator. Plastic-adherent monocytes were incubated with granulocyte-macrophage-colony-stimulating factor (10 ng/mL; Miltenyi Biotec, Bergisch Gladbach, Germany) in macrophage serum-free medium (Gibco-Life Technologies, Carlsbad, CA, USA) to generate primary human macrophages. After 6 days, macrophages were harvested using 1 mM EDTA/PBS (Sigma-Aldrich, St. Louis, MO, USA). If indicated, macrophages and autologous PBMCs were co-cultured in a 1:5 ratio in a poly-L-lysine-coated 8-chamber slide.

### 5.3. Protein Expression and Cell Lysis

The plasmids coding for ^His_^eGFP_C3bot, ^His_^eGFP_C3bot_E174Q_, ^His_^eGFP (for its origin, see [23]), C3bot, C3bot_E174Q_, ^Cys_^C3bot_E174Q_ (for origin see [52]), C2I, or C2IIa were heat-shock transformed into competent *Escherichia coli* BL21. For the first preculture, 5 mL LB medium (1% tryptone, 0.5% yeast extract, 1% NaCl, 100 µg/mL ampicillin) was inoculated with a single colony for 5–8 h at 37 °C and at 180 rpm in a shaking incubator. A second 150 mL overnight culture in an Erlenmeyer flask was inoculated with the preculture. The main culture of 4 L LB medium was inoculated with the overnight preculture (30 mL per L) and incubated at 37 °C and 180 rpm until the OD_600_ reached 0.6–0.8. Protein expression was induced by adding 0.5 mM isopropyl β-D-1-thiogalactopyranoside (IPTG, Carl Roth, Karlsruhe, Germany). Subsequently, the incubation temperature was decreased to 16 °C (for eGFP-labeled proteins) or to 29 °C (GST-tagged proteins) for the main culture incubated at 180 rpm overnight. For harvesting, the cells were centrifuged at 5500 rcf and 4 °C for 10 min, and the pellet was resuspended in 40 mL buffer. GST-Lysis buffer (10 mM NaCl, 20 mM Tris, 1% Triton X-100, 1% phenylmethylsulfonyl fluoride (PMSF); pH 7.4) was used for GST-tagged proteins and NPI-20 buffer (50 mM NaH_2_PO_4_, 300 mM NaCl, 20 mM imidazole, 1% PMSF; pH 8.0) for His-tagged proteins. The cells were lysed by sonication (10 pulses each for 20 s with intermediate pauses of 30 s). Cell fragments were removed by centrifugation at 13,000 rcf and 4 °C for 30 min. The supernatant was filtered through 0.45 µm and 0.2 µm syringe filters.

### 5.4. Purification of GST-Tagged Proteins

C3bot, C3bot_E174Q_, C2I, and ^Cys_^C3bot_E174Q_ were purified as GST-tagged proteins, as described previously [23]. The filtered cell lysates were incubated overnight at 4 °C with 1.2 mL Protino Glutathione Agarose 4B-beads (Macherey-Nagel., Düren, Germany), which were preequilibrated in PBS (137 mM NaCl, 2.7 mM KCl, 8 mM Na_2_HPO_4_, and 1.8 mM KH_2_PO_4_; pH 7.4). After centrifugation at 3000 rcf for 5 min, the beads were washed three times (twice with washing buffer (150 mM NaCl, 20 mM Tris–HCl; pH 7,4) and once with PBS). The proteins were eluted by removing the GST-tag with thrombin (80 NIH units, Amersham Biosciences, Little Chalfont, GBR) for 1 h at RT. The beads were removed by centrifugation for 30 s at 10,000 rcf and 4 °C. Thrombin was removed by incubating the supernatant with 120 µL Benzamidine–Sepharose 6B-beads (GE Healthcare, Chicago, IL, USA) for 10 min at RT. Centrifugation at 10,000 rcf and 4 °C for 30 s was used to remove the benzamidine beads, and the concentration of purified proteins was determined in SDS-PAGE by comparison to a BSA standard.

### 5.5. Purification of His-Tagged Proteins

The proteins ^His_^eGFP_C3bot, ^His_^eGFP_C3bot_E174Q_, and ^His_^eGFP were produced as described previously [23]. C2II was produced as a His-tagged protein, as described in [45]. In general, filtered cell lysate containing the His-tagged proteins was incubated with preequilibrated (in NPI-20, see Section 5.3) PureCube 100 INDIGO Ni-agarose (Cube Biotech, Monheim am Rhein, Germany) overnight at 4 °C. PureCube 1-step batch Midi Plus Columns (Cube Biotech, Monheim am Rhein, Germany) was used to collect the beads, and they were washed three times with 20 mL NPI-20. For elution of the His-tagged proteins, NPI-250 (50 mM NaH_2_PO_4_, 300 mM NaCl, 250 mM imidazole; pH 8.0) was used. Protein fractions were analyzed in SDS-PAGE, and the ones with the highest amount of target protein and the lowest content of impurities were collected. The buffer was exchanged with PBS by ultrafiltration (Vivaspin20 with 10 kDa molecular weight cutoff, Merk, Darmstadt, Germany). The protein solutions were stored at −80 °C, and the concentration of purified proteins was determined via SDS-PAGE by comparison to a BSA standard.

### 5.6. Coupling of Maleimide-Labeled Cargo to ^Cys_^C3bot_E174Q_

The modular ^Cys_^C3bot_E174Q_ system was loaded with maleimide-labeled cargo by incubation at 4 °C for 2 h. For mDL488 (Thermo Scientific, Waltham, MA, USA), ^Cys_^C3bot_E174Q_ and cargo were mixed at a 1:1 molar ratio. Due to the steric hindrance of PEG for mPEG_FITC (Nanocs, Boston, MA, USA), a 1:50 molar ratio (^Cys_^C3bot_E174Q_ to cargo) was needed. mC2I was prepared by labelling C2I at 4 °C for 2 h with a 10-fold molar excess of MBS (Thermo Scientific, Waltham, MA, USA) followed by removal of unbound MBS with Zeba Spin Desalting Columns (7 kDa molecular weight cutoff, Thermo Scientific, Waltham, MA, USA). For generation of mC2I-^Cys_^C3bot_E174Q_, ^Cys_^C3bot_E174Q_ and mC2I were mixed at a 1:1 molar ratio. Subsequent to coupling of the cargo, free maleimide-labeled small-molecule cargo (mDL488 and mPEG_FITC) was removed by two rounds of buffer exchange via the Zeba Spin Desalting Columns with 7 kDa molecular weight cutoff protocol. Finally, the concentration of loaded transporters was determined via SDS-PAGE by comparison to a BSA standard.

### 5.7. Flow Cytometry

Human-monocyte-derived macrophages were detached with 1 mM EDTA/PBS for 20 min at 37 °C, while lymphocytes were harvested by centrifugation (1300 rpm for 10 min). Per sample, either 2 × 10^5^ macrophages or lymphocytes were incubated with ^His_^eGFP or ^His_^eGFP_C3bot_E174Q_, or left untreated for 20 min at 37 °C. Afterwards, cells were washed with FACS buffer (1% FCS (Biochrom, Berlin, Germany) and 0.1% sodium azide (VWR, Radnor, PA, USA) in PBS (Gibco-Life Technologies, Carlsbad, CA, USA) and centrifuged for 10 min at 1300 rpm. The supernatant was discarded, and cells were incubated directly before measurement for 1 min with 50 µg/mL trypan blue to quench extracellular eGFP signals as described in [40]. Intracellular fluorescence was detected using a FACSCalibur™ flow cytometer (BD Biosciences, Heidelberg, Germany)**.** Data analysis was performed using Flowing Software 2.5.1 (Turku Bioscience, Turku, Finland).

### 5.8. Phase Contrast Microscopy

Cells were seeded in 96-well microtiter (Corning Incorporated, Corning, NY, USA) plates and incubated with the indicated test substance at 37 °C and 5% CO_2_ on the next day. One phase contrast image was taken per well (three wells for each treatment) with a LEICA DMi1 microscope connected to a MC 170 HD camera (both Leica Microsystems, Wetzlar, Germany). Representative images are depicted with scale bars.

### 5.9. Cell Viability and Proliferation Assay

Cells were seeded in a 96-well microtiter plate and treated as indicated. After the indicated incubation time, 10 μL CellTiter 96 AQueous One solution was added, containing 3-(4,5-dimethylthiazol-2-yl)-5-(3-carboxymethoxyphenyl)-2-(4-sulfophenyl)-2H-tetrazolium (MTS). Cells were incubated further for 1–2 h, and the absorbance at 492 nm was measured in a plate reader.

### 5.10. STED Super-Resolution Microscopy

STED microscopy was performed as described in [23,40]. A total of 10^5^ monocyte-derived macrophages were seeded per well in an 8-well µ-slide with a glass bottom (ibidi GmbH, Gräfelfing, Germany). The cells were incubated with 250 nM of the indicated protein (^His_^eGFP, ^His_^eGFP_C3bot, or ^His_^eGFP_C3bot_E174Q_) for 30 min at 37 °C. Afterwards, the cells were washed twice with cold PBS and fixated with 3.2% paraformaldehyde in PBS (32% PFA aqueous solution, Electron Microscopy Sciences, Hatfield, PA, USA) for 20 min at RT. After washing the cells three times with PBS, the cells were permeabilized and blocked in 3% BSA and 0.3% TritonX-100 in PBS for 2 h. The samples were incubated overnight with 1 µg/mL of primary rabbit anti-EEA1 antibody (Thermo Scientific, Waltham, MA, USA) and 0.5 µg/mL Atto594-conjugated GFP-booster nanobody (Chromotek, Planegg-Martinsried, Germany) in 1:10 diluted blocking solution at 4 °C. The cells were washed three times and incubated with 1 µg/mL of the secondary Atto647N-conjugated goat anti-rabbit antibody (Sigma-Aldrich, St. Louis, MO, USA) dissolved in 1:10 diluted blocking solution. Unbound antibodies were removed by washing the samples three times with PBS. Before imaging, PBS was exchanged with 2,2′-thiodiethanol (97% solution in PBS, pH 7.5). A self-build dual-color 3D STED microscope [53] was used for image recording with an average power of 0.8 µW for each excitation beam and 1.3 mW for each depletion beam. The pixel size was 12.5 nm, and images were captured with 300 µs dwell time and approximately 150 counts as a typical peak photon number. The recorded pictures were analyzed with ImageJ (v1.52n, National Institute of Health, Bethesda, MD, USA). A Gaussian blur σ = 1 pixel and >20 count intensity threshold was applied for better visualization.

The eGFP signals and their co-localization with EEA1 signals were automatically quantified by a self-written search algorithm in Python 3.7. The algorithm loaded respective raw image file pairs, i.e., one image file containing Atto594-conjugated GFP-booster nanobody signal intensities and the belonging second image file representing signal intensities for EEA1. On both image files, a Gaussian blur σ = 1 pixel was applied to reduce background noise and to smoothen signals for later automated search. Next, a threshold of >35 counts was set for eGFP signal intensities and >50 counts for EEA1 signal intensities to further eliminate unwanted background. After thresholding, the algorithm horizontally searched for eGFP signals. Whenever a signal was found horizontally, the respective vertical coordinate was searched for. Each coordinate pair was saved for later use and counted. Thereby, for each donor, the mean signal number per image was calculated and averaged for five individual blood donors (n = 5). After every pixel was analyzed with regard to eGFP signals, the saved eGFP coordinate pairs were loaded to be compared with the image file containing EEA1 signal intensities. In more detail, within a radius of 250 nm (based on an estimated endosome diameter of 500 nm) from the previously found eGFP coordinate pair, the algorithm searched for EEA1 signals, indicating an EEA1-associated eGFP signal. Such co-localizing signals were successively counted. EEA1 signals above a radius of 250 nm were cut off as non-co-localizing eGFP signals. For each donor, the mean percentage of co-localizing eGFP signals was calculated (co-localizing eGFP signals divided by the total number of eGFP signals) and averaged for five individual blood donors (n = 5).

### 5.11. Immunofluorescence Staining for Confocal or Epifluorescence Microscopy

Cells were incubated as indicated at 37 °C in 5% CO_2_. Afterwards, cells were washed twice and fixed (4% PFA, Sigma-Aldrich, St. Louis, MO, USA). For MHC class II staining, cells were incubated with 2% BSA in PBS and labeled with anti-HLA-DR antibody (1:200, L243, Leinco, St. Louis, MO, USA) for 30 min at RT. After three washing steps with PBS, MHC class II was detected by Cy5-conjugated goat anti-mouse antibody (1:250, Dianova, Hamburg, Germany). If indicated, cell nuclei were stained either with DAPI (1:200, Sigma-Aldrich, St. Louis, MO, USA) diluted in 1% BSA and 0.1% Triton X-100 in PBS, or with 5 µg/mL Hoechst33342 in PBS for 10 min at RT. Confocal images were acquired by using the inverted laser scanning confocal microscope LSM 710 (Zeiss, Oberkochen, Germany). Epifluorescence microscopic images were recorded using the iMIC digital microscope (FEI, Munich, Germany). Images were processed using ImageJ software (v1.51n, National Institute of Health, Bethesda, MD, USA).

### 5.12. SDS-PAGE and Western Blotting

For protein separation depending on molecular weight, SDS-PAGE was used with 12.5% acrylamide gels. Subsequently to electrophoresis, the proteins were transferred onto a nitrocellulose membrane by using semi-dry blotting, which was controlled by Ponceau S (AppliChem GmbH, Darmstadt, Germany) staining. Unspecific binding to the membrane was blocked by incubation in 5% skim milk powder diluted in PBS-T (137 mM NaCl, 2.7 mM KCl, 8 mM Na_2_HPO_4_, 1.8 mM KH_2_PO_4_, 0.1% Tween20; pH 7.4) for 1 h at RT. After washing with PBS-T, the membrane was incubated with the indicated antibody or streptavidin–peroxidase conjugate (1:5000; Sigma-Aldrich, St. Louis, MO, USA) diluted in PBS-T for 1 h at RT. For eGFP detection, 1:10,000 diluted anti-GFP antibody (ab290, Abcam, Cambridge, GBR) was used. HSP90 was detected with 1:500 diluted HSP90 α/β antibody (F-8, Santa Cruz Biotechnology, Dallas, TX, USA), while for EEA1-detection, 1:1000 diluted rabbit EEA1 polyclonal antibody (PA1-063A, Thermo Fisher Scientific, Waltham, MA, USA) was used. Unbound antibodies/proteins were removed with three washing steps with PBS-T for 5 min at RT on an orbital shaker. For the detection of eGFP and EEA1, 1:2500 diluted mouse anti-rabbit IgG-HRP (sc-2357, Santa Cruz Biotechnology, Dallas, TX, USA) was used. For detection of HSP90, 1:2500 diluted m-IgGκ BP-HRP (sc-516102, Santa Cruz Biotechnology, Dallas, TX, USA) was used. The peroxidase-labeled antibodies/proteins were detected with Pierce ECL Western blotting substrate (Thermo Fisher Scientific, Waltham, MA, USA) and X-ray films (AGFA Health Care, Mortsel, BEL).

### 5.13. Sequential ADP-Ribosylation Assay

Cells were seeded in 24-well microtiter plates and treated as indicated at 37 °C and 5% CO_2_. Extracellular toxins were removed by washing the cells two times with PBS. The medium was removed, and the samples were frozen at −20 °C and thawed in ADP-ribosylation buffer (20 mM Tris-HCl, 1 mM EDTA, 1 mM DTT, 5 mM MgCl_2_, cOmplete (1:50, freshly added); pH 7.5) to lyse the cells. The samples were collected, and 5 pmol fresh C3bot and 6-biotin-17-NAD^+^ (10 µM) were added in excess. The sequential ADP-ribosylation reactions at 37 °C were started and stopped at the same time (after 30 min). Laemmli buffer (0.3 M Tris-HCl, 10% SDS, 37.5% glycerol, 0.4 mM bromophenol blue) was added, and the samples were heat denatured. Notably, in this sequential ADP-ribosylation reaction, only the non-ADP-ribosylated Rho can be biotin-labeled from 6-biotin-17-NAD^+^. Hence, in SDS-PAGE and Western blotting (see Section 5.12), the non-ADP-ribosylated Rho in intact cells is detected with streptavidin–peroxidase conjugate. For densitometric analysis of the detected protein bands, ImageJ software (v1.51n, National Institute of Health, Bethesda, MD, USA) was used. Importantly, weak signals detected in this assay indicate strong toxin activity in intact cells.

### 5.14. Digitonin-Based Cell Fractionation Assay

A total of 10^6^ U-DCS cells were seeded on a 24-well microtiter plate. After two days, the cells were incubated with the respective proteins (^His_^eGFP_C3bot, ^His_^eGFP_C3bot_E174Q_, and ^His_^eGFP) with indicated concentrations and time points at 37 °C. Subsequently, the cells were carefully washed twice with PBS and then incubated with digitonin (20 µg/mL in PBS) for 5 min at RT. Thereby, the cell membrane was permeabilized, and the cytosol left the cells through the previously formed pores during incubation for 25 min at 4 °C on ice. In this process, the cells were divided into two fractions: the supernatant (cytosol-only fraction) and the solid cellular portion (membrane fraction) containing cellular organelles, vesicles, cell membranes, and the remaining cytosolic proteins that did not flow out through the pores. The fluorescence of eGFP was analyzed in both fractions using a microplate reader at 488 nm excitation and 510 nm emission. SDS-PAGE and Western blot analysis (see Section 5.12) were performed to ensure clean separation of both fractions. The early endosomal marker EEA1 was only detectable in the membrane fraction. Cytosolic HSP90 can be detected in both fractions since it is not possible to force complete outflow of all cytosolic proteins. The presence of ^His_^eGFP and ^His_^eGFP-labeled proteins was analyzed in the respective cell fractions with the GFP antibody.

### 5.15. Data Analysis and Visualization

The depicted data points are provided as mean ± standard deviation (± SD) with corresponding sample size (n). A two-tailed unpaired Student’s *t*-test was used to compare the two treatment groups. The following significance levels were defined: not significant (ns) *p* > 0.05, * *p* < 0.05, ** *p* < 0.01, *** *p* < 0.001, **** *p* < 0.0001. Diagrams were generated by using GraphPad Prism (version 9.1.2 GraphPad Software, San Diego, CA, USA). Figures were assembled in Inkscape (version 0.92, Free Software Foundation, Boston, MA, USA).

## Data Availability

The data presented in this study is available on request from the corresponding author.

## Supplementary Materials

The following supporting information can be downloaded at https://www.mdpi.com/article/10.3390/toxins14100711/s1, Table S1. Quantification of the mean eGFP signals per image given for the individual human blood donors. Table S2. Co-localization analysis of eGFP and EEA1. Table S3. Quantification of the cytosol fraction from the digitonin-based cell-fractionation assay. Table S4. Quantification of the membrane fraction from the digitonin-based cell-fractionation assay. Figure S1. Cell-type selective uptake of ^His_^eGFP_C3bot_E174Q_ into primary human macrophages compared to lymphocytes ex vivo. Figure S2. Quantification of eGFP-positive macrophages from Figure 2b. Figure S3. Detection of the eGFP-labeled C3bot variants in SDS-PAGE and Western blotting. Figure S4. The modular thiol–maleimide system enabled attachment of cargo molecules to ^Cys_^C3bot_E174Q_. Figure S5. Internalization of mPEG_FITC into macrophages and DCs was strongly enhanced by coupling to ^Cys_^C3bot_E174Q_. Figure S6. The cell viability/proliferation of U-DCS cells was not or only minimally effected by the transported mC2I.
