# Supplementary material for: Clostridium botulinum C3 Toxin for Selective Delivery of Cargo into Dendritic Cells and Macrophages
Source: Toxins (Basel). 2022 Oct 18;14(10):711. doi: 10.3390/toxins14100711 (PMC9610094; doi:10.3390/toxins14100711)
Supplement: Supplementary file 1 [file toxins-14-00711-s001.zip › toxins-1923592-supplementary.pdf]

toxins

MDPI

Supplementary material

Clostridium botulinum C3 toxin for selective delivery of cargo into dendritic cells and macrophages

Maximilian Fellermann [1], Mia Stemmer [1], Reiner Noschka [2], Fanny Wondany [3], Stephan Fischer [1], Jens Michaelis [3], Steffen Stenger [2] and Holger Barth [1,*]

[1] Institute of Experimental and Clinical Pharmacology, Toxicology and Pharmacology of Natural Products, University of Ulm Medical Center, 89081 Ulm, Germany

[2] Institute for Medical Microbiology and Hygiene, University of Ulm Medical Center, 89081 Ulm, Germany.

[3] Institute of Biophysics, Ulm University, 89081 Ulm, Germany

* holger.barth@uni-ulm.de

Uptake Analysis of eGFP into Early Endosomes of Human Macrophages

Table S1. Quantification of the mean eGFP-signals per image given for the individual human blood donors. The eGFP-signals of the STED super resolution images were quantified as described under 5.10 in the materials and methods section. The mean eGFP-signals per image are given for each individual donor (1-5) and form the basis for the column diagram in Figure 1b.

donor number	NC	^{His}-eGFP_C3bot$_{E174Q}$	^{His}-eGFP_C3bot	^{His}-eGFP
1	0.0	29.5	18.7	0.0
2	0.0	49.7	49.7	0.0
3	0.0	81.0	77.0	2.8
4	0.0	9.3	19.3	0.3
5	0.0	84.0	13.5	0.0
mean ± SD	0.0 ± 0.0	50.3 ± 28.9	35.6 ± 24.3	0.6 ± 1.1

Table S2. Co-localization analysis of eGFP and EEA1. As described in the material and methods section under 5.10, it was analyzed whether the counted eGFP signals (see Table S1) were detected in close proximity (250 nm) with the early endosomal marker EEA1. The number of eGFP-signals in close proximity to EEA1 was divided by the total number of eGFP-signals and the percentage is given in the table. If no eGFP-signals were detected, this is indicated with "not quantifiable (nq)". Note: The given co-localization percentage tells nothing about the absolute number of detected eGFP-signals and does therefore not correlate with amount of uptake. This is especially important for ^{His}-eGFP since here only very few eGFP-signals were detected for only two of the tested five donors (see Table S1).

donor number	NC [%]	^{His}-eGFP_C3bot$_{E174Q}$ [%]	^{His}-eGFP_C3bot [%]	^{His}-eGFP [%]
1	nq	98.7	98.9	nq
2	nq	74.1	91.6	nq
3	nq	86.8	90.8	96.7
4	nq	58.4	89.8	100.0
5	nq	91.7	86.7	nq
mean ± SD	nq	81.9 ± 15.9	91.6 ± 4.5	98.3 ± 2.4

^{His}-eGFP_C3bot$_{E174Q}$ is Selectively Internalized into human Macrophages ex vivo.

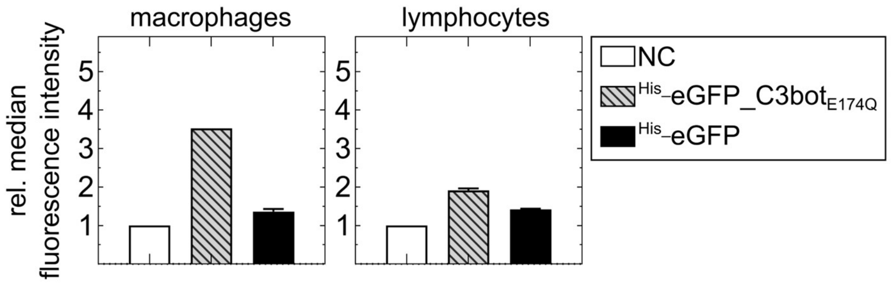

Figure S1. Cell type-selective uptake of ^{His}-eGFP_C3bot$_{E174Q}$ into primary human macrophages compared to lymphocytes *ex vivo*. Human monocyte-derived macrophages and lymphocytes of the same donor were separately treated with 250 nM ^{His}-eGFP_C3bot$_{E174Q}$ or ^{His}-eGFP for 20 min at 37 °C. The cells were washed with PBS and extracellular or membrane bound eGFP-signals were quenched with trypan blue directly before flow cytometry measurement. The averaged relative median fluorescence intensity (normalized to untreated cells (NC)) is depicted in the column diagrams (mean ± SD, n = 2).

Quantification of eGFP-Positive Human Macrophages in Co-Culture with Lymphocytes ex vivo

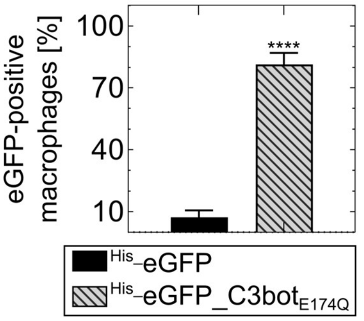

Figure S2. Quantification of eGFP-positive macrophages from Figure 2b. Monocyte-derived macrophages and lymphocytes of the same human blood donor were co-cultured as described in Figure 2b. The percentage of macrophages which internalized ^{His}-eGFP or ^{His}-eGFP_C3bot$_{E174Q}$ was quantified by manually counting the number of eGFP-positive macrophages and dividing it through the total number of macrophages. For each treatment, five images were analyzed from one representative experiment (n = 5). Compared to the ^{His}-eGFP-treatment, statistical significance was tested via Student's t-test and columns are labeled according to following significance levels: ns $p > 0.05$, * $p < 0.05$, ** $p < 0.01$, *** $p < 0.001$, **** $p < 0.0001$.

Detection of the eGFP-Labeled C3bot Variants

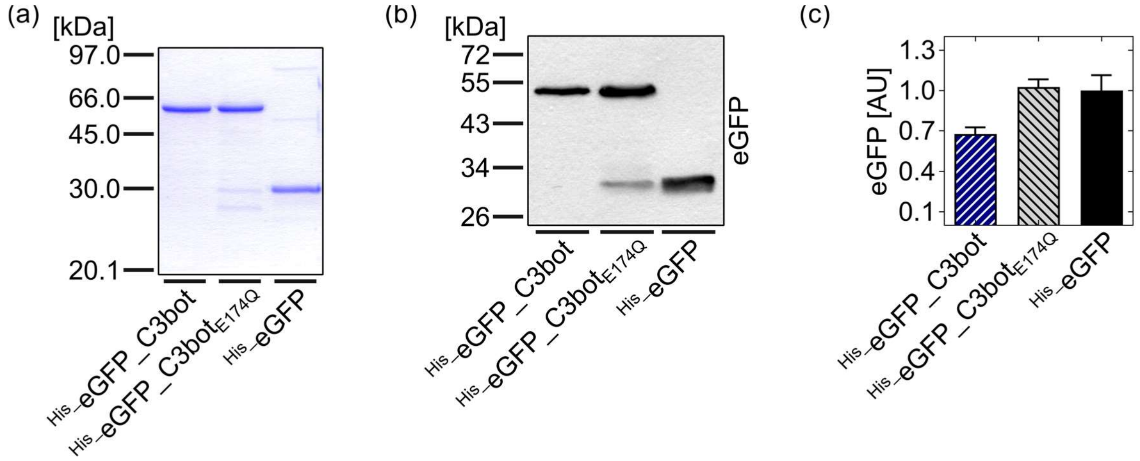

Figure S3. Detection of the eGFP-labeled C3bot variants in SDS-PAGE and Western blotting. (a) 2 µg of the eGFP-proteins (His_eGFP_C3bot, His_eGFP_C3botE174Q and His_eGFP) were analyzed by SDS-PAGE and Coomassie brilliant blue R250 staining. (b) The eGFP-label of the proteins (^{His}-eGFP_C3bot, ^{His}-eGFP_C3bot$_{E174Q}$ and ^{His}-eGFP) was detected by Western blotting after loading 2 µg of each onto a SDS-gel. (c) The in (b) detected integrated density values for eGFP were normalized to the ^{His}-eGFP control. (b, c) eGFP was detected by Western blotting independent of the attachment of C3bot or C3bot$_{E174Q}$. For ^{His}-eGFP_C3bot the signal intensity was slightly reduced.

Quantification of the Digitonin-Based Cell-Fractionation Assay (Western Blot)

Table S3. Quantification of the cytosol fraction from the digitonin-based cell-fractionation assay. U-DCS cells were treated like described for Figure 5a. The Western blots were quantified and for the cytosol fraction the detected eGFP-signals was divided by the detected HSP90-signals. The ratios of the calculated integrated density values are given for each repetition individually. If no eGFP-signals were detected this is indicated by "nd". The calculated values for each reception were averaged and given in the last row as mean ± SD.

repetition number	NC [AU]	^{His}-eGFP_C3bot$_{E174Q}$ [AU]	^{His}-eGFP_C3bot [AU]	^{His}-eGFP [AU]
1	0.00 (nd)	1.56	1.52	0.03
2	0.00 (nd)	6.59	7.55	0.75
3	0.00 (nd)	5.85	5.75	0.16
4	0.00 (nd)	0.38	7.39	0.49
5	0.00 (nd)	4.68	2.47	1.51
6	0.00 (nd)	7.63	4.03	0.29
7	0.00 (nd)	1.49	1.64	0.58
mean ± SD	0.00 (nd)	4.02 ± 2.86	4.34 ± 2.60	0.54 ± 0.49

Table S4. Quantification of the membrane fraction from the digitonin-based cell-fractionation assay. U-DCS cells were treated like described for Figure 5a. The Western blots were quantified and for the membrane fraction the detected eGFP-signals was divided by the detected EEA1-signals. The ratios of the calculated integrated density values are given for each repetition individually. If no eGFP-signals were detected this is indicated by "nd". The calculated values for each reception were averaged and given in the last row as mean ± SD.

repetition number	NC [AU]	^{His}-eGFP_C3bot$_{E174Q}$ [AU]	^{His}-eGFP_C3bot [AU]	^{His}-eGFP [AU]
1	0.00 (nd)	2.90	1.39	0.00 (nd)
2	0.00 (nd)	0.79	1.61	0.33
3	0.00 (nd)	0.98	0.47	0.07
4	0.00 (nd)	1.77	1.90	0.04
5	0.00 (nd)	2,17	1.94	0.41
6	0.00 (nd)	0.52	3.91	0.14
7	0.00 (nd)	1.99	1.36	0.20
mean ± SD	0.00 (nd)	1.59 ± 0.86	1.80 ± 1.05	0.17 ± 0.15

Attachment of Cargo to the Modular Thiol-Maleimide System

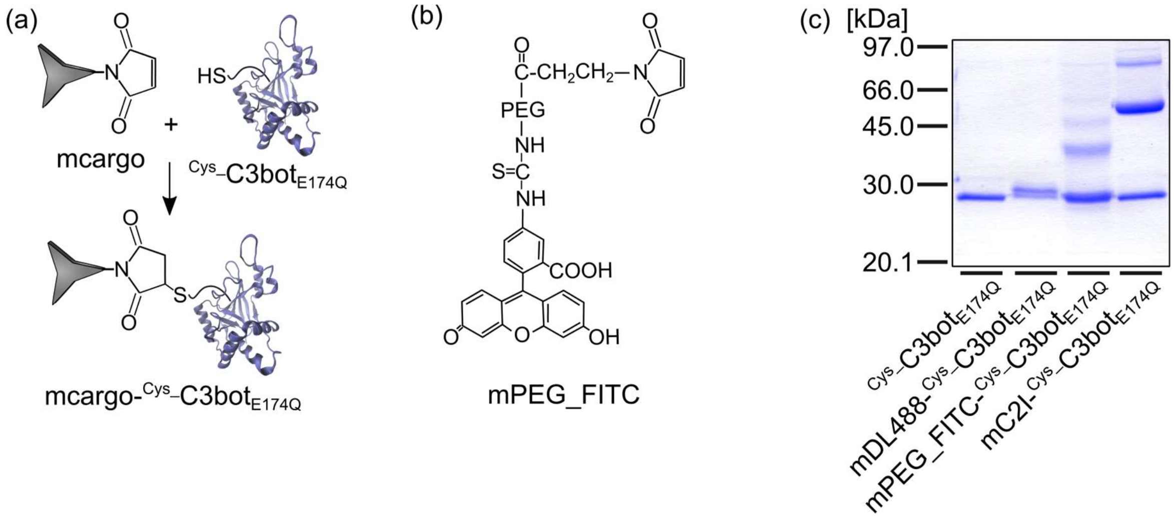

Figure S4. The modular thiol-maleimide system enables attachment of cargo molecules to ^{Cys}-C3bot$_{E174Q}$. (a) The cartoon depicts the attachment of maleimide-labelled cargo molecules to ^{Cys}-C3bot$_{E174Q}$ with free thiol (HS-) group. In ^{Cys}-C3bot$_{E174Q}$, the first amino acid

was mutated to a cysteine (A1C) for providing a functional thiol group. (b) The molecular structure of mPEG_FITC is depicted as example for a maleimide-labelled cargo molecule. For our experiments we used mPEG_FITC with a molecular weight of 5 kDa. (c) Different cargo molecules were attached to ^{Cys}-C3bot$_{E174Q}$ as indicated by a shift to higher molecular weight (MW is dependent on respective cargo). For mDL488, this shift was relatively small since the fluorophore has a MW of only 0.8 kDa. For attached mPEG_FITC (5 kDa) and mC2I (50 kDa), bigger shifts were detected. However, free ^{Cys}-C3bot$_{E174Q}$ and uncoupled cargo was visible, indicating incomplete labeling reaction. Due to these contaminations with free cargos, the concentrations in control-treatments were increased as indicated (see Figure 7 and Figure S5)

Uptake of mPEG_FITC into Target Cells is Strongly Enhanced by the ^{Cys}-C3bot$_{E174Q}$ Transport System

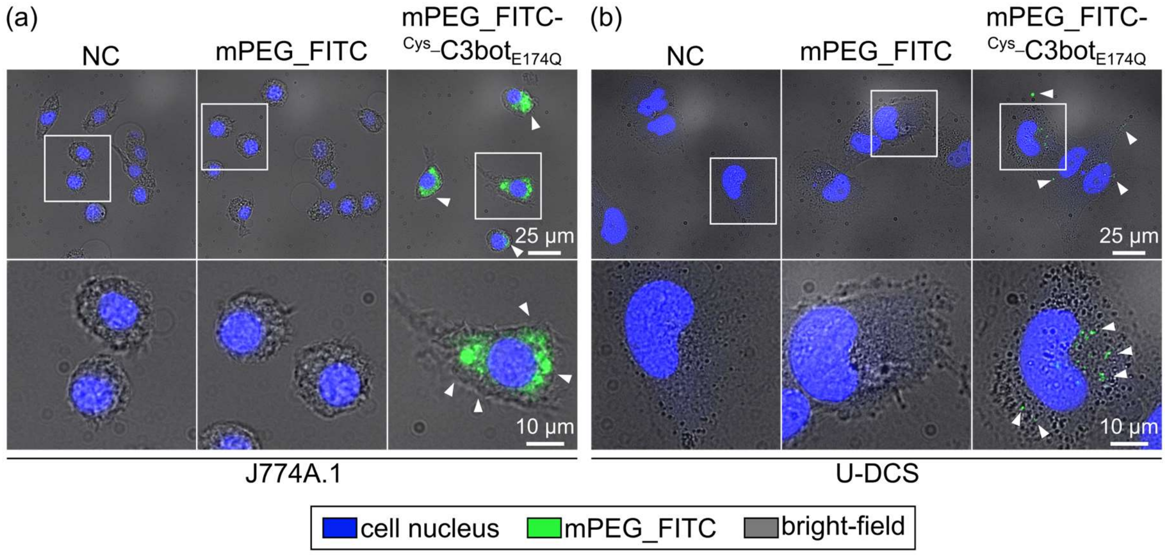

Figure S5. Internalization of mPEG_FITC into macrophages and DCs is strongly enhanced by coupling to ^{Cys}-C3bot$_{E174Q}$. J774A.1 macrophages (a) and U-DCS cells (b) were treated with 5 µM mPEG_FITC alone, 100 nM of coupled mPEG_FITC-^{Cys}-C3bot$_{E174Q}$ or with medium only (NC) for 30 min at 37 °C. Subsequently, the cells were washed twice with PBS and fixated. The cell nuclei were stained with 5 µg/mL Hoechst 33342 in PBS and epifluorescence microscopy was performed. The detected mPEG_FITC-signals (green) were merged with the stained nuclei (blue) and the recorded brightfield image (gray tones). In the brightfield images, cell shape and membrane borders were visible to confirm that all detected mPEG_FITC-signals are indeed internalized into the cell. Representative images per sample are depicted with magnification (white squares and lower row) and scale bars (25 µm and 10 µm for the magnification). Note: Since the detected mPEG_FITC-signal were more intense for J774A.1 cells, the images were processed individually for each cell line and should therefore not be directly compared. The figure is modified from [37] under author rights.

Transported mC2I Causes Cell Rounding But only Minimally Affects Cell Viability/Proliferation within 50 h

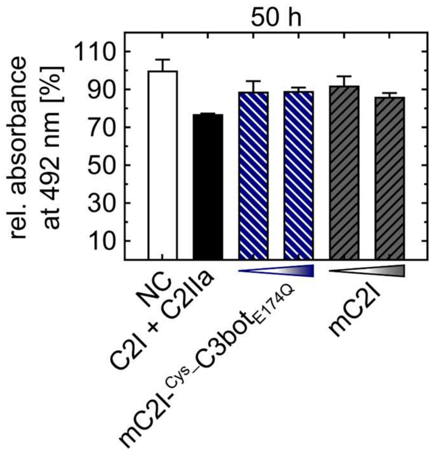

Figure S6. The cell viability/proliferation of U-DCS cells is not or only minimally effected by the transported mC2I. U-DCS cells were treated with 50, or 200 nM mC2I-^{Cys}-C3bot$_{E174Q}$, 220 or 880 nM mC2I, a combination of 1 nM C2I with 1.66 nM C2IIa, or left untreated (NC). After 50 h of incubation, the cells were incubated for 1-2 h with MTS for performing a cell viability/proliferation assay. The

relative absorbance at 492 nm per treatment is depicted in the column diagram as mean ± SD (n = 3) for a representative experiment. Note: The cargo molecule C2I causes cell rounding (see Figure 7) by ADP-ribosylation of G-actin but does not directly affect the cell viability as described in literature [44, 45].
